# Open Source Drug Discovery in Practice: A Case Study

**DOI:** 10.1371/journal.pntd.0001827

**Published:** 2012-09-20

**Authors:** Christine Årdal, John-Arne Røttingen

**Affiliations:** 1 Section for Global Health, The Norwegian Knowledge Centre for the Health Services, Oslo, Norway; 2 Department of Health Management and Health Economics, Institute for Health and Society, University of Oslo, Oslo, Norway; 3 Department of Global Health and Population, Harvard School of Public Health, Boston, Massachusetts, United States of America; McGill University, Canada

## Abstract

**Background:**

Open source drug discovery offers potential for developing new and inexpensive drugs to combat diseases that disproportionally affect the poor. The concept borrows two principle aspects from open source computing (i.e., collaboration and open access) and applies them to pharmaceutical innovation. By opening a project to external contributors, its research capacity may increase significantly. To date there are only a handful of open source R&D projects focusing on neglected diseases. We wanted to learn from these first movers, their successes and failures, in order to generate a better understanding of how a much-discussed theoretical concept works in practice and may be implemented.

**Methodology/Principal Findings:**

A descriptive case study was performed, evaluating two specific R&D projects focused on neglected diseases. CSIR Team India Consortium's Open Source Drug Discovery project (CSIR OSDD) and The Synaptic Leap's Schistosomiasis project (TSLS). Data were gathered from four sources: interviews of participating members (n = 14), a survey of potential members (n = 61), an analysis of the websites and a literature review. Both cases have made significant achievements; however, they have done so in very different ways. CSIR OSDD encourages international collaboration, but its process facilitates contributions from mostly Indian researchers and students. Its processes are formal with each task being reviewed by a mentor (almost always offline) before a result is made public. TSLS, on the other hand, has attracted contributors internationally, albeit significantly fewer than CSIR OSDD. Both have obtained funding used to pay for access to facilities, physical resources and, at times, labor costs. TSLS releases its results into the public domain, whereas CSIR OSDD asserts ownership over its results.

**Conclusions/Significance:**

Technically TSLS is an open source project, whereas CSIR OSDD is a crowdsourced project. However, both have enabled high quality research at low cost. The critical success factors appear to be clearly defined entry points, transparency and funding to cover core material costs.

## Author Summary

Open source drug discovery can be an influential model for discovering and developing new medicines and diagnostics for neglected diseases. It offers the opportunity to accelerate the discovery progress while keeping expenditures to a minimum by encouraging incremental contributions from volunteer scientists. Publishing raw data and results in the public domain is positive within the context of neglected diseases since it facilitates open collaboration while obviating the ability to patent any results. In this way it effectively de-links the research and development costs from the sales price of the end product, the new medicine or diagnostic. This case study demonstrates that implementations of the open source model can differ while still achieving the ultimate goal of obtaining high quality research at reduced costs. However, the importance of clearly defined entry points, transparency and funding are shared success factors. These findings present the practical challenges of implementing a theoretical concept and hopefully will assist other scientists in organizing future open source drug discovery projects.

## Introduction

The vast majority of drug research and development (R&D) performed globally is directed towards the needs of high-income countries [1]. The former Global Forum for Health Research and the work that led to its establishment asserted that 90% of all health R&D investment is spent on areas that concern only 10% of the world's population [2]–[4]. High-income countries have the resources to pay, either publicly or privately, a price which gives the innovator a profitable return on investment. The problem, of course, is that the medical needs of high-income countries are not the same as low-income countries. There are a host of diseases that are primarily endemic to low-income countries, diseases like dengue fever, malaria and schistosomiasis. Incentivizing R&D investments by standard incentives like patents simply does not produce the greatly needed, new medicines or diagnostics for these diseases (which are often labeled “neglected”). These are neglected because the market does not offer sufficient purchasing power. This market failure is an internationally recognized problem and has been a major focus of the World Health Organization (WHO).

In 2003 a Commission on Intellectual Property Rights, Innovation and Public Health was established under the auspices of WHO in order to apprise appropriate funding and incentive mechanisms for these neglected diseases. A number of initiatives have resulted from the Commission's recommendations including the formation of an expert working group to suggest and evaluate options to incentivize R&D for these diseases [5]. A large variety of financing and coordinating mechanisms have been proposed. One that has received some support is open source drug discovery.

Open source drug discovery is a model based upon the open source movement within the computer software industry. Basically it takes two primary attributes, namely the collaboration of volunteers and free access to the results, and applies them to drug discovery. This should ultimately translate into new drugs entering the market at prices determined by generic competition.

The concept has been discussed within the academic literature for almost a decade. One of the first proposals by Maurer, Rai and Sali [6] laid out the concept and applied it particularly to tropical diseases. Subsequently, there have been several high-level descriptions of example projects [7], [8] and more recently, empirical examples [9] of models, methods, processes and tools. However, the literature has not united behind a single, comprehensive definition of the concept. This combined with the multitude of titles given to the concept (e.g. precompetitive collaboration, data sharing, open access R&D, etc.) makes a common understanding difficult.

Luckily, the non-profit research organization, Results for Development Institute (“R4D”), has recently undertaken a high-level review of open source drug discovery projects aimed at neglected diseases [10]. One of the results of this review is a straightforward definition. R4D defines a set of attributes that a project must comply with in order to be considered open source:

1. The project's data must be open access, meaning that anyone can view the data free-of-charge.
2. The project must provide a forum for open collaboration (across organizational and geographical boundaries).
3. The project must be governed by a set of rules that mandates the project's “openness”.

If a project adheres to all three requirements, the resulting advantages should be: verified content, collaborative projects, the creation of a commons of knowledge and reduced costs for the project (resulting in lower prices for the end product).

In an open source project data is made publicly-available for anyone and everyone to verify. In drug discovery this means that all virtual and laboratory results are published with as much of the raw data available as possible. This should include enough data for someone knowledgeable in the topic to review and critique the data.

Collaboration across organizational and geographical boundaries offers several benefits. If enough researchers can be incentivized to collaborate, even small contributions by many researchers can significantly progress a project. It also opens a project to new external ideas and approaches. It is anticipated that the majority of the researchers will contribute on a volunteer basis, thereby reducing the cost of the project.

A commons of knowledge is knowledge that is owned by the public, meaning that there is no individual owner. All sciences contain vast commons of knowledge. For example, in mathematics, algebra, geometry and calculus are all a part of the commons of knowledge. No one owns them; they are public knowledge. These knowledge commons grow when researchers place their data in the public domain. This is most commonly done by publishing the data without first patenting it. Knowledge residing in the public domain may not be patented since novelty is required to patent. This means that anyone can use, distribute and further develop the research without paying a royalty to, or even notifying, the innovator. If all the data necessary to manufacture a new drug are placed in the public domain, anyone may undertake the necessary regulatory steps for approval and begin to manufacture the drug.

In open source computing it is more common to utilize specialized licenses rather than the public domain since software code is most commonly protected by copyright which is awarded automatically. These licenses allow the innovator to maintain some level of control over the innovation, generally ensuring that attribution is given and that the code is freely accessible for anyone to redistribute and modify. Any license in compliance with the Open Source Definition [11] is considered open source. These same aims can also be achieved by pairing a patent with a standard license allowing free use of the patent so long as the use adheres to a set of conditions. Examples include instances where innovators allow patented medicines to be manufactured by producers in low-income countries for local use only (i.e. equitable licenses).

Project costs of open source projects are significantly reduced based upon the percentage of work performed by volunteers as well as the absence of the administrative costs that accompany contract creation and royalty payment. Since the research is placed in the public domain, the price of the manufactured product is essentially de-linked from the cost of the R&D. Manufacturers set a price point based solely upon their own costs and expectations of the market's willingness to pay. Ideally generic competition is introduced immediately.

Three similar concepts (open access, open innovation and crowdsourcing) are often confused with open source. Open access means that anyone can view, copy or distribute some form of content (e.g. an article, book, etc.) free-of-charge; it does not permit changing the content [12].

Open innovation is simply the use of external sources of R&D [13]. This may include paying royalties to the innovator and does not necessitate any type of transparency or commons formation and is therefore not related to the general “open definition”. For example, AstraZeneca recently agreed that a certain set of external scientists could access all of the data related to approximately 20 experimental drugs that they have stopped researching. This data is not open to the public. These drugs are under patent, and AstraZeneca will commercially benefit if the scientists manage to determine a profitable use of these molecules [14]. Open innovation offers the potential benefits of collaborative projects and reduced costs of both the project and the end product, but does not offer verified content or the creation of a commons of knowledge.

“Crowdsourcing” is the use of volunteers to perform a specified task, generally through an open call [15]. For example, the FoldIt game has players fold proteins into their most chemically stable configuration, introducing new possibilities to the scientists who gather and research the players' efforts [16]. The contributors do not own their output, and crowdsourced outputs may or may not be protected by intellectual property rights. Crowdsourcing offers the same benefits of open innovation - collaborative projects and reduced costs of both the project and the end result, but does not necessarily offer verified content or the creation of a commons of knowledge.

Open source is an important model for neglected diseases R&D because it offers the opportunity to accelerate the discovery progress while keeping expenditures to a minimum. Patents in these instances are neither desired nor justifiable since the cost of patenting will likely exceed any potential profits.

A current gap within the academic literature is detailed profiles and evaluations of ongoing open source initiatives for neglected-disease research. This is the objective of our case study – to learn from the first movers of open source drug discovery, their successes and failures, in order to generate a better understanding of how a much discussed theoretical concept actually works in practice. After a search for relevant cases, we have studied two cases in detail: The Council for Scientific and Industrial Research Team India Consortium's Open Source Drug Discovery project (CSIR OSDD) and The Synaptic Leap's Schistosomiasis project (TSLS).

The objective of the case study is to answer the following research questions:

- How do existing open source drug discovery initiatives attract volunteers, create a collaborative model, achieve progress, address the need for physical supplies and manage intellectual property rights?
- What have these projects accomplished to date?

Our results demonstrate that open source drug discovery initiatives can make significant achievements. However, there is no one formula for success. Critical success factors are clearly defined entry points, transparency and funding to cover all material costs.

## Methods

A case study was chosen to research open source drug discovery projects in-depth in accordance with pre-defined research questions. Yin [17] recommends a case study approach when the researcher wants to answer “how” and “why” questions, when an experiment is inappropriate or when it is necessary to understand the context in greater detail. He categorizes case studies as either explanatory (attempting to find the causality of a specific case), exploratory (exploring an intervention with no clear outcome) or descriptive (describing a real-life phenomenon and its context). We decided to conduct a descriptive case study to examine the real-life phenomenon of open source drug discovery as it applies to neglected disease R&D.

### Case Selection

We chose open source drug discovery projects targeted towards neglected diseases that have had at least one year of continuous data from multiple individuals. We identified twelve potential cases of an open source approach to drug discovery, mainly through our ongoing research of the topic but also through other articles reviewing the topic [8]–[10]. The potential cases identified along with their conformance to the selection criteria are given in Table 1.

**Table 1:** Potential Cases for Inclusion.

Potential Case	Collaboration Efforts Viewable	Project Status	Timeframe of Data
The Council for Scientific and Industrial Research Team India Consortium's Open Source Drug Discovery project (CSIR OSDD)	Yes	Active	2008 – Ongoing
Collaborative Drug Discovery	No	Active	2004 – Ongoing
Cambia's Open Innovation	No	Inactive	2009
PATH's Malaria Vaccine Initiative	No	Active	1999 - Ongoing
Structural Genomics Consortium	No	Active	2003 - Ongoing
The Pool for Open Innovation against Neglected Tropical Diseases	No	Inactive*	2009–2011
The Synaptic Leap's Malaria Project	Yes	Inactive**	2006–2008
The Synaptic Leap's Schistosomiasis Project (TSLS)	Yes	Active	2006 - Ongoing
The Synaptic Leap's Toxoplasma Project	Yes	Inactive	2006–2007
The Synaptic Leap's Tuberculosis Project	Yes	Inactive	2006–2007
Tropical Diseases Initiative	No	Active	2004 - Ongoing
TDR Targets	No***	Active	2007 - Ongoing

* This project has been transformed to the WIPO Re:Search project.

** This project has been restarted in 2012 with a considerable amount of activity.

*** TDR Targets does share posted lists. However, these are not collaboration efforts towards a designated goal.

Two cases fit our selection requirements: The Council for Scientific and Industrial Research Team India Consortium's Open Source Drug Discovery project (CSIR OSDD) and The Synaptic Leap's Schistosomiasis Project (TSLS). The other potential cases were excluded either because the project's collaboration efforts were not viewable (meaning that data was shared but the process of producing the data was not shared or collaboratively performed) or the project was inactive (meaning that a small number of individuals would occasionally make a posting which was most often an interesting article about the topic).

### Data Collection

Data were gathered from four sources: an analysis of the cases' websites, interviews of participating members, a survey of potential members of CSIR OSDD and a literature review. Additionally the project managers of both cases were sent our findings, and their comments have been incorporated into this paper.

All websites of the two projects have been reviewed focusing on aspects of collaboration and progress. The licenses have also been reviewed to understand how intellectual property is managed.

Telephone and written interviews were performed from November 2010 to April 2011. Interview content focused on collaboration, intellectual property and progress. An interview template was devised and reviewed by two external researchers familiar with open source drug discovery (Annex S1). We posted introductions to our case study on both the CSIR OSDD and TSLS websites, asking interested individuals to e-mail us if interested in participating. We also directly e-mailed participants where we could find contact information (n = 99). Fourteen (14) individuals responded, representing both project leaders and active members. Among the 14, only ten completed all interview topics and this was disproportionately members of TSLS project (n = 9). The individual completing the interview from the CSIR OSDD project had observed the project but not contributed. However, four CSIR OSDD project members partially completed the interview.

A survey (Annex S2) of potential members of the CSIR OSDD project was performed in February and March 2011. The CSIR OSDD project was selected because they are performing general tuberculosis drug discovery activities where as the TSLS project is performing a very specific development task in regards to making a new synthesis of a known molecule, making it more difficult to identify researchers with similar research interests. PubMed was searched on January 31, 2011 for articles published within the last year containing the phrase “*Mycobacterium tuberculosis* genome”. A second search was performed on February 10, 2011 for articles published within the last year containing the phrase “Tuberculosis drug discovery”. The searches resulted in 221 and 112 articles respectively. The corresponding author's e-mail address was retrieved from each of these articles and then duplicates were removed. Sixty-one individuals completed the survey (n = 46 from the genome group and n = 15 from the drug discovery group).

A literature review was performed to identify any academic articles relevant to our research questions. This was done by searching Google Scholar on December 6, 2011 with the following strings, achieving the following results:

- “The Synaptic Leap”+schistosomiasis (n = 15)
- CSIR India “open source drug discovery” (n = 48)

These articles were read.

### Ethics Statement

We sought approval for our research portfolio (including interviews and surveys) from the Norwegian Committees for Medical and Health Research. The Committee decided that our research did not require their ethical approval since we are studying collaboration amongst scientists and not patients. With that said, all interview participants were informed orally that their interview responses would be treated confidentially and that their participation was completely voluntary. Written consent was deemed unnecessary since interview participants responded individually to a call for interviews from a website posting. The survey data were analyzed anonymously. The interview data were analyzed in combination with the scientists' postings on publicly-available websites.

## Results

We evaluated the two cases in regards to four aspects: accomplishments, process (including attracting volunteers, collaboration and addressing the need for physical supplies), management of intellectual property, progress and funding. We will present the two cases separately.

### CSIR's Open Source Drug Discovery Project

The Council for Scientific and Industrial Research Team India Consortium's Open Source Drug Discovery project (CSIR OSDD) started in 2008 with an initial grant from the Government of India of approximately US $35 million (of which US $12 million has been released to date). Their vision is “*to provide affordable healthcare to the developing world by providing a global platform where the best minds can collaborate & collectively endeavor to solve the complex problems associated with discovering novel therapies for neglected tropical diseases like Malaria, Tuberculosis, Leshmaniasis, etc.*” Initially they have targeted tuberculosis as their primary research area (see Table 2).

**Table 2:** CSIR's Open Source Drug Discovery Project at a glance.

Focus:	Tuberculosis medicines (all aspects of discovery and development)
Year started:	2008
Funding:	INR 1.5 billion (∼US $35 million), the Government of India
Number of contributors:	451
License:	An original license, “OSDD Terms and Conditions”
Achievements to date:	(1) Curated a re-annotation of *Mycobacterium tuberculosis* genome which generated a metabolome and protein-protein functional network to be used to identify potential drug targets (2) Generated 11 models for prediction of anti-tuberculosis activity (3) Created a chemical repository of small molecules
Number articles publishing the project's scientific findings:	Five [21], [22], [38]–[40]
Evaluations/audits:	None known

### Accomplishments

CSIR OSDD aims to discover novel therapies for tuberculosis. Its activities are spread throughout every stage of the discovery process (from drug target identification to lead optimization). It has 54 molecules in process and has initiated discussions with pharmaceutical companies regarding pre-clinical and clinical trials. Its main achievements to date are: the re-annotation of the *Mycobacterium tuberculosis* genome and the generation of 11 models for prediction of anti-tuberculosis activity [18].

The genome of the *Mycobacterium tuberculosis* strain H37Rv was first published in 1998 [19]. Since publishing, new research has been performed in such areas as gene functionality, associated proteins, interactions and potential drug targets. Most of this research is available electronically but on many different websites. Data curation involves establishing and developing long-term repositories of reference data [20]. The CSIR OSDD project created a data repository for genome-level information regarding the strain H37Rv, by recruiting volunteers to gather relevant research articles, extract the data and transcribe it into a standardized format. The aggregation of this process is TBrowse, a publicly-available integrative genomics map, http://tbrowse.CSIR OSDD.net/ [21]. The formation of TBrowse demonstrated that students could successfully contribute to open source drug discovery. With this proof of concept performed, CSIR OSDD moved onto a more complex task called Connect to Decode, annotating the tuberculosis genome. Again students collated the data contained in published articles regarding the approximate 4,000 genes contained in the tuberculosis genome. For those genes whose function was unknown, participants computationally extrapolated the possible function(s). This work created a metabolome (a complete set of small molecules involved in growth, development and reproduction) and protein-protein functional network for *Mycobacterium tuberculosis* that is being used to identify potential drug targets. This data is contained on website called Sysborg.

Eleven groups have worked independently to develop models for prediction of anti-tuberculosis activity. Two of these models have been published [22] and the other nine are in the process of being written up. CSIR OSDD has purchased the virtual screening data of 20,000 molecules, where 140 of these molecules have shown promising anti-tubercular properties. CSIR OSDD has built a new repository [23] (the OSDD Chemical Database) to gather data on these and other promising molecules. As of February 22, 2012, 304 molecules reside in the virtual repository, submitted by 17 individuals. Four molecules have been screened against tuberculosis, 14 against malaria.

To perform these accomplishments, CSIR OSDD has created a significant amount of infrastructure. They utilize several websites including:

- A publicly-available informational website (www.osdd.net) that describes the project in general and gives links to the other websites. We will call this the “CSIR OSDD website” from now forward.
- An online collaboration forum (http://sysborg2.osdd.net) requiring a username and password to access any content. This includes pages to enter and view ideas, projects, laboratory notebooks and documents. A project management system allows members to track tasks and progress. A forum tracks all comments. A resources page allows participants to request new biological or chemical materials as well as financing. A community page allows members to group into activity-related communities. Members can check that they have received due credit on an attribution page. An eLearning page links members to online tutorials to assist them with their contributions. There is also social networking functions – messaging, linking with friends, blogging and a calendar. We will call this website “Sysborg” from now forward. This website replaced a previous wiki-style website in 2010. The content from this initial website is no longer accessible/viewable. Users have expressed that the new Sysborg website is more difficult to navigate and has technical problems. CSIR OSDD is working on improving the website based upon the users' feedback.
- The publicly-available CRDD web portal (http://crdd.osdd.net/) provides access to numerous drug discovery computing tools throughout the phases of drug discovery (including target identification, virtual screening and drug design) as well as the OSDD Chemical Database.
- Several publicly-available forums (http://groups.google.com/group/osdd-public, http://osddnews.blogspot.com/, http://scienceopenscience.blogspot.com/, http://twitter.com/osdd) which are used for general (non-task-related) discussions.

### Process

According to a description of the project [24], the workflow follows a standard process comprised of the following steps:

1. Projects or ideas are posted by any community member on Sysborg.
2. The community then reviews the project/idea.
3. A principal investigator (mostly experienced scientists) will take responsibility for the project/idea and secure any necessary funding from CSIR OSDD.
4. The community collaborates on the project and produces results (typically in the form of laboratory notebooks).
5. The results are made available on Sysborg for the community to review. Members provide input on project monitoring and quality control.
6. An unstated, but practiced, next step is that the results are published in a peer-reviewed journal.

#### Step 0 - Logging onto the website

Before an individual can browse Sysborg, he/she must register and await an automatically generated password to log on. However, with these details a user can only access the social functions of the website, not any of the project data. The e-mail states, “*Please note that the team will verify your details and it takes approximately 2–3 working days to assign you necessary permissions to access the portal content*.” Once these permissions are granted the user may access the majority of functionality within Sysborg.

#### Step 1 - Posting a project or an idea to Sysborg

As of November 30, 2011 there were 52 ideas and 139 projects posted on Sysborg, although the reporting section of Sysborg states that there are 99 ideas and 523 projects. We are uncertain if this means that some content is hidden or that the reporting system is in error.

Projects typically include a problem description and work plan. Most projects (92% as of December 6, 2011) have designated a project manager. There are on average two members per project, although 45% of projects have no project members. Projects may be associated with comments, ideas, laboratory notebooks or other projects. There is no status associated with a project so it is unclear if a project is pending, in progress or completed. There is a link within each project to a project management system, but this system seems not to be in use.

#### Step 2 - Project review

The second procedural step is that the community reviews the project. This appears to happen rarely on Sysborg (however, the CSIR OSDD project management team has informed us that many of the existing projects were reviewed in the previous website but this review has not been migrated to Sysborg). Out of 139 projects, 80 (58%) had no comments associated with them. Ten projects (7%) had three or more comments (with a median of four comments but one project with 34 comments).

#### Step 3 - Secure funding

The third step is that a project manager will take responsibility for the project and secure any necessary funding from CSIR OSDD. From the CSIR OSDD website it appears that named institutions have responsibility for tasks within the drug discovery process, e.g. National JALMA Institute for Leprosy & Other Mycobacterial Diseases has responsibility for identifying drug targets through biological repositories and strains, the Institute of Genomics and Integrative Biology in Delhi has responsibility for identifying drug targets through *Mycobacterium* tuberculosis annotation, etc., although other institutions are encouraged to participate [18].

Before funding may be secured, a project must be peer reviewed. After all questions from the peer-review have been answered, the project and its budget are reviewed by a committee of three specified individuals. If the committee recommends the budget, the funds are released [25]. This peer review and approval process is rarely visible in Sysborg; we found only two examples where all activities were visible [26], [27].

There is also an automated resources request process which includes cash requests among other resources (e.g. genomic DNA materials). This process appears to be rarely used.

#### Step 4 - Attracting contributors and collaborating

The fourth step is that the community collaborates on the project and produces results. As mentioned above, CSIR OSDD partners with institutions that have specific responsibilities. Eight CSIR India laboratories and 36 Indian universities and academic institutes [28] were selected through a screening process including on-site inspections [29]. Upon selection, it appears that the institutions receive funding to cover the costs of equipment, chemicals and consumables for CSIR OSDD contributors [30].

Researchers from these institutions become project managers, leading and organizing activities. The Project Director contacts project managers directly to instigate new activities, or project managers may suggest new activities. Students and other researchers are encouraged to participate through open calls for contributions. Students reported through the interviews hearing about CSIR OSDD through direct contact, the Internet and word-of-mouth. Students also reported through the interviews that they were highly motivated to help fellow Indians by finding cures for tuberculosis. Learning new skills was also a motivation.

CSIR OSDD has published several articles detailing the aims of the project [31], [32], likely to draw attention to the project from other tuberculosis researchers. We were curious to know if tuberculosis researchers worldwide were aware of the project and if they ever viewed the data. We surveyed the corresponding authors (n = 298) of all articles contained within PubMed, published in the last year focused on either tuberculosis drug discovery or *Mycobacterium tuberculosis* genome. We received 61 responses (20% response rate). Thirteen authors (or 21%) were aware of TBrowse (the publicly-available integrative genomics map) and of those seven had viewed TBrowse.

As of February 16, 2012 there were 5,444 users registered in Sysborg. Of these, 451 had accrued points (as reported by CSIR OSDD management). Points are awarded after the completion of a specified task [33], however we could not find any data specifying how the point value is calculated. By accruing points, contributors can achieve higher levels of membership which gives the contributor greater rights, privileges and responsibilities [34]. In some instances, contributors can receive monetary rewards [33].

Students may need to apply to contribute to resource-constrained activities. For example, in one project students have applied and been selected to utilize grid-based supercomputing facilities from their desktops. Before students are given access to this facility, they must complete an application form and affidavit stating that “*all activities performed, including raw data and results would be the property of the [CSIR OSDD] community to be shared with the community and covered under the [CSIR OSDD] License Terms and conditions of use*.” [35] This application form is sent via e-mail to the CSIR OSDD Technical Committee for approval with a copy sent via surface mail. To train the students in using this functionality, a three-day boot camp was held in Calicut for about 35 participants where travel costs were paid for. The presentations from this boot camp were filmed and placed on YouTube [36]. Additionally a large amount of training materials have been made available on Sysborg, in YouTube and a telephone-based help desk has also been set up [26].

Project managers are not only responsible for recruiting contributors but also creating assignments (sometimes with deadlines), giving instruction, ensuring that the necessary facilities and materials are present, performing quality assurance, and following up that assignments are received [37]. Laboratory notebooks contain the data for all laboratory tasks. There were 363 laboratory notebooks as of November 30, 2011. These notebooks were largely consolidated to a few projects; five projects had three or more lab notebooks with one project having 119. Most projects (n = 110 or 79%) had no associated laboratory notebooks.

According to an interviewee, after each activity is completed, a group meeting is held either face-to-face or via Skype to go through the results and finalize the data. The final results are then posted to Sysborg. This may explain why 95% of all laboratory notebooks have no associated comments.

#### Step 5 - Peer-review of results

Results posted to Sysborg are to be reviewed by the community as a part of quality control. The CSIR OSDD website states that all project managers report directly to the Project Director online, a core team meets monthly and the chief mentor reviews the progress of the platform quarterly along with the board of mentors [34]. We could find no evidence on Sysborg of this review process.

#### Step 6 – Publishing

Lastly, project results may be published in a peer-reviewed journal. Five articles [21], [22], [38]–[40] have been published to date covering the results of the project's collaborative tuberculosis drug discovery activities, four of these in 2011 alone, a significant achievement. CSIR OSDD has also published two articles describing the CSIR OSDD process [31], [32] and one regarding tools [9]. The CSIR OSDD website also lists other scientific publications that have received funding from CSIR OSDD but are not the result of project collaboration [41]–[43].

### Management of Intellectual Property

No content may be viewed on Sysborg without first logging on. When registering, the user must accept the terms and conditions of the CSIR OSDD license, a non-standard license written specifically for the project [44]. The license affirms that CSIR OSDD owns all content posted to Sysborg (§3.1). Therefore, content is not a part of the public domain. All improvements based upon data within Sysborg must be contributed back to CSIR OSDD under a worldwide royalty-free non-exclusive license (§3.5–6). There is no stipulation in the license that CSIR OSDD must adopt non-exclusive licensing of the resulting products or any stipulations regarding the final price of these products. However, the mission states clearly that they aim “*to make available affordable medicines to every single person of the developing world*.”

### Progress

CSIR OSDD has mapped out a process for discovering and developing new tuberculosis medicines. They have 54 molecules in the pipeline, including two candidates in the hit to lead phase which are being optimized in collaboration with private partners (which seem to follow the same overall process) [24]. They have instigated talks with pharmaceutical industry to perform the preclinical and clinical trials. Their approach to clinical trials is to build facilities specifically for clinical trials within publicly-funded hospitals. These trials would be conducted by CSIR OSDD in combination with the hospital personnel and experts from private pharmaceutical companies. All data will be made available (presumably anonymized) [24]. We found no evidence of clinical trials on Sysborg so we presume that these are planning activities in anticipation of forthcoming trials.

### Funding

The government of India has committed to grant CSIR OSDD INR 1.5 billion (or about US $35 million) of which US $12 million has already been paid out [33]. These funds pay the administrative costs of the project including equipment and material costs at the partner institutions and the salaries of a few contributors. Most work is done by unpaid volunteers. However, the project does hire individuals at times to perform specific tasks. For example, 20 female scientists are planned (or have been) hired to work from their homes for four hours a day [30]. Expert mentors are paid to attend meetings [30]. Vacancies are regularly posted on the website for paid positions such as project assistants [45].

### The Synaptic Leap's Schistosomiasis Project

The Synaptic Leap website was launched in 2006 with an aim “*to provide a network of online research communities that connect and enable open source biomedical research*” [46]. It was launched with four pilot disease research areas: malaria, schistosomiasis, toxoplasma and tuberculosis. Each area had a project leader with the responsibility of gathering and motivating international researchers to contribute to the Synaptic Leap community by sharing results, giving feedback and possibly undertaking new research tasks. Since launch, the malaria, toxoplasma and tuberculosis communities have been relatively silent. However, the schistosomiasis community has consistently utilized the website to share findings, discuss research results and identify new, necessary research tasks (see Table 3).

**Table 3:** The Synaptic Leap's Schistosomiasis Project at a glance.

Focus:	Development of a low-cost synthesis of an existing schistosomiasis drug, praziquantel
Year started:	2006
Funding:	AUS$315,000 (∼US $330,000) Australian government and WHO
Number of contributors:	37
License:	Scientific discoveries in the public domain and copyright according to the Creative Commons Attribution 2.5 License
Achievements to date:	Produced the schistosomiasis drug, praziquantel, in enantiopure form
Number articles publishing the project's scientific findings:	One [52]
Evaluations/audits to date:	None known

### Accomplishments

The aim of the TSLS project was a well-defined drug development task – to generate the off-patent schistosomiasis drug, praziquantel, as a single enantiomer. This would remove the bitter taste of the original drug making it more palatable for children as well as remove some of its side effects. This has been needed for years but companies would not invest, likely because the innovation was not suitably lucrative since an inexpensive drug already existed. Additionally the patent on praziquantel expired in the 1990s [47], and the needed change was likely not sufficiently novel to warrant a new patent. The optimization of praziquantel had long been a high priority of WHO which was affirmed in TDR's (Special Programme for Research & Training in Tropical Diseases) Scientific Working Group on Schistosomiasis in 2005 and repeated in its Business Plan of 2008–2013. [48] This led to the funding of the TSLS project in 2008 by both WHO and the Australian government. The TSLS project completed this task in 2011.

To perform these accomplishments, TSLS has made use of web tools that were already available such as The Synaptic Leap website and an open source online laboratory notebook [49]. The laboratory notebook was chosen because it allowed contributors to enter scientific data more easily than The Synaptic Leap website.

### Process

Dr. Matthew Todd became the leader of the schistosomiasis project in 2006. He was already working on the problem of the production of praziquantel as a single enantiomer but wanted the project to go faster than typical academic speed. He thought that open source might be a solution to attract industry participation. The project was first discussed on the TSLS website in January 2006 [50]. However, even though Todd regularly updated the website, there was little external interest shown in the project. From 2006 to 2008 there were 35 postings initiated on the website, with only four of these coming from individuals other than Todd.

In 2008 the project received their funding (although contracting delays resulted in the laboratory work actually not starting until January 2010). This allowed the project to hire a full-time postdoctoral researcher and cover laboratory expenses for Ph.D. students, mentored by Todd at the University of Sydney. This gave the project some needed momentum. From project initiation in 2006 until project funding in the beginning of 2010, 10% of new postings were initiated from external contributors (those not a part of Todd's team at the University of Sydney). After the funding was received 30% of postings were made by external contributors. However, comments posted by external contributors did not vary significantly (increasing only from 50% to 53%). At the time of funding, significant external marketing efforts were also undertaken (see below).

The data from the on-going experiments were regularly posted in the online publicly-available laboratory notebook [49] and summarized on The Synaptic Leap, without peer review. Todd did not want to slow the speed of sharing the data by implementing an offline peer review process. He expected project contributors to give the researchers feedback, and this turned out to be the case. Key findings have received as many as 14 comments; entries average 1.5 comments each, with 50% of all new postings receiving comments. This process has been an adjustment for some of the contributors. There were concerns that mistakes would be published with name attribution. One researcher stated that he used more time to check his results before publishing them online. Ultimately, Todd expected peer review to be done through publishing, and two articles summarizing the results of this project have been published in September and October 2011 (one with a focus on the project results and one focused on the process) [51], [52].

In order to make contributions as easy as possible, Todd regularly posted an update on TSLS detailing progress and descriptions of the next tasks needed [53], [54]. This minimized the time that potential contributors needed to sift through backdated postings to come up to speed. It also avoided duplication of efforts. The project did not have an official project plan or deadlines, but it was time-constrained by funding parameters (three years).

Even after the postdoctoral researcher was hired to contribute, there was a hope that greater external interest could be raised for the project. Todd began giving speeches including a Google TechTalk in April 2010 [55]. After each article, blog and presentation, the project experienced significant increases in website traffic [51]. It was also decided to reach out to a closed chemistry networking forum on LinkedIn. This positively resulted in 20 comments from 11 different scientists, new to the project, and four private e-mails [51]. One of the respondents was a Dutch contract research organization interested in participating in the project [51]. This was an important milestone for the project because the CRO had the equipment and expertise to perform some of the necessary tasks very quickly (they completed tasks in weeks as opposed to the months it would probably have otherwise taken). This industry-academic support enabled the project to complete the project before the funding ran out.

Ninety-seven (97) individuals have registered on the Synaptic Leap indicating that they are actively participating or are interested in participating in research for schistosomiasis. Thirty-seven (37) contributed to the TSLS project. The contributors include six members of Todd's team, four industry representatives, 15 academics/researchers, one retiree, two informatics professionals, and 9 of unknown affiliation. Contributors were based in Africa, Europe, Oceania and North America. Only one postdoctoral researcher from the University of Sydney was paid specifically to work on the project. Motivations for participation included accelerating own research, intellectual stimulation, signaling abilities and a belief in the benefits of open collaboration. Their contributions ranged from one-off comments regarding the project to substantial postings regarding laboratory results.

### Management of Intellectual Property

TSLS places all scientific discoveries in the public domain, therefore, obviating the ability to patent them. All of the website content is copyright protected according to the Creative Commons Attribution 2.5 License unless otherwise stipulated [56]. All content may be viewed without a username and password. If an individual wants to make a posting on the Synaptic Leap website, he/she can either leave a comment as a guest or as a registered user. A guest must supply a valid e-mail address which is not viewable with the comment. Registering requires a username and e-mail address. An automated system sends a log-on password. There is no requirement to accept a license at time of registration.

Intellectual property does not play a major role in this project since a version of praziquantel has been in the public domain for almost two decades.

### Progress

The scope of this project was limited to a specific problem. Once they managed to generate a single enantiomer of praziquantel, the expectation was that the project would be complete (although the project continues looking at more elegant solutions to the problem). The next steps of scaling up the modified drug to commercial quantities and any regulatory approvals needed would be performed externally by a pharmaceutical manufacturer in partnership with WHO.

### Funding

Funding was important to the project because it allowed for the recruitment of a full-time postdoctoral researcher whose postings provided fresh, regular content giving the project momentum. The grant money paid the salary of the postdoctoral student, all administrative supplies and covered the cost of shipping the samples to any interested laboratory. Contributing organizations did not receive any monies from the project.

### Comparative Analysis: CSIR OSDD and TSLS

Firstly, we would like to acknowledge that both cases have made great accomplishments in meeting their aims. CSIR OSDD has persuaded a large number of volunteers to contribute and published four articles in 2011, a significant accomplishment for a group of volunteers. TSLS has gathered contributors from around the globe, both from academia and the private sector and has managed to fulfill its goal.

The two cases operate very differently and differ greatly in magnitude. CSIR OSDD is a vast project, encouraging international collaboration on its website, but in actuality, geared principally towards Indian researchers and students. The funding from the Indian government applies only to activities within India [24]. There are many workshops and face-to-face meetings in India as well as private e-mail correspondence between teacher and pupil. This, in essence, translates into an Indian-centric project. TSLS, on the other hand, has attracted contributors internationally, albeit substantially fewer than CSIR OSDD, with a variety of motivations. Both have obtained funding used to pay for access to facilities, physical resources and, at times, labor costs. TSLS releases its results into the public domain, where as CSIR OSDD asserts ownership over its results.

If we return to R4D's definition of open source – the application of open access, open collaboration and open rules – it is useful to analyze each case's adherence to the definition in order to understand the impact of this adherence (see Table 4).

**Table 4:** Are the cases open source?

	Open Access	Open Collaboration	Open Rules
CSIR OSDD	Yes, but only with significant effort	No	No
TSLS	Yes	Yes	Yes

CSIR OSDD's scientific research results are placed on Sysborg which requires a user to log on before any content may be viewed. The content is not searchable through general search engines like Google. Technically, the content is open access because a username and password are eventually granted to users allowing them to view the data free-of-charge. However, we believe that this tight control of the data is actually a barrier to entry. Most potential contributors will want to browse the website before contributing, and they may lose interest in the two days or more that it takes to receive access to the full content. Indeed, a few TSLS contributors reported through the interviews that they had tried to access CSIR OSDD and had given up in frustration. CSIR OSDD' process limits contributors to only those who have a strong motivation to contribute.

CSIR OSDD has assigned certain tasks to partner institutions. This is likely a practical solution to achieving progress. These institutions receive funding and have commitments back to CSIR OSDD. They must follow an agreed structure and process. Other institutions or individuals can no doubt assist in any activity. However, since much of the process is opaque (through face-to-face meetings, Skype or private e-mail) and not reported back through Sysborg, open collaboration is difficult. This opaqueness does not promote cross-organizational or geographical linkages. Until the processes and decision-making are made more transparent and easier to follow on Sysborg, CSIR OSDD does not fit the definition for open collaboration.

CSIR OSDD's license awards the project ownership over all data. Data may not be used by other entities without entering into a contract with CSIR OSDD. The license may also be considered viral since all improvements based upon CSIR OSDD data are to be granted back to CSIR OSDD (i.e. future generations of improvements are subject to the CSIR OSDD license if any of the original CSIR OSDD data was used). This may make industry shy away from participating in the project. CSIR OSDD has taken a very protective approach of its data likely so that it is not expropriated and exploited by a third party. This is understandable considering the potential commercial value of new tuberculosis medicines. However, CSIR OSDD's license does therefore not mandate “openness”. CSIR OSDD states that the project will shepherd its new products up through regulatory approval and then make them available to the generic drug industry without any exclusivity [24]. It is unclear whether they intend to patent the drugs and offer a non-exclusive license to generic manufacturers, utilize the public domain or an alternative intellectual property strategy. Perhaps they have not yet decided themselves. The license language, however, does not mandate openness.

We believe that rather than a strictly defined open source project, CSIR OSDD is actually a highly successful crowdsourcing project, using volunteers to perform specified and structured tasks. They have achieved most of the advantages of open source identified by R4D. The data results are verified (although offline), but the project's impressive publishing demonstrates that its work has passed peer review muster. The contributions of 400+ volunteers result in a significant cost savings. Undoubtedly, any medicines that they develop will enter the market at a low price point. They have not, however, succeeded in creating open collaboration or a public commons of knowledge. They have created a proprietary knowledge repository.

TSLS is largely in adherence to the open source drug discovery definition. All of TSLS' data are publicly-available without a password. Searches within Google for related TSLS content return all of TSLS' related websites. This makes it easy for potential contributors to firstly find the project and then browse the content to get a feeling for the project. However, TSLS' website could also be improved. Postings are not necessarily in chronological order and there is no easy method to see all postings related to one disease area. Thanks to TSLS' project manager's continuous efforts to summarize the current state of play, these inconveniences are minimized.

TSLS' websites allow for open collaboration across organizational and geographical boundaries. It is stressed that e-mails should be avoided. Raw data is placed directly on the website awaiting virtual peer review. Observers can easily follow the threads of the process.

TSLS uses well known legal concepts with the public domain and a creative commons license. Both mandate “openness”. Results may be utilized by third parties without contracts or royalties.

TSLS has achieved all of the open source advantages. Its content is transparently verified on the website with the additional peer review of publishing in top-ranked journals. The data forms a knowledge commons. Global collaboration was achieved between representatives from both academia and industry. The grant funding and volunteer contributions of industry significantly sped up the progress of the project, achieving cost savings.

## Discussion

These two cases demonstrate that drug innovation can be performed using an open source approach, albeit in very different ways and not necessarily in strict adherence to the definition of open source drug discovery. Adherence to the definition is not necessarily that important. As a crowdsourced project, CSIR OSDD has still achieved great success by persuading volunteers to perform high quality research at low cost, which, of course, is the goal of open source collaboration. The definition is still useful though, to evaluate how different projects approach transparency, collaboration and access to results, but not necessary to spur on high quality, low cost drug discovery. The cases do point to three common critical success factors: clearly defined entry points, transparency and funding.

Both projects attracted volunteers by publicizing the respective projects through descriptive articles in academic journals and utilizing social media and networks. CSIR OSDD has also effectively paired up with Indian universities and colleges, incentivizing students to volunteer as parts of classroom assignments or positioning participation as valuable hands-on experience. They have also built in an element of patriotism, linking finding cures for tuberculosis as an Indian responsibility due to the high prevalence of tuberculosis in India. This patriotic effect is reinforced through project marketing efforts, like the project's music video [57]. The entry point into CSIR OSDD is through the classroom which is likely to limit international participation in the project. Rather TSLS' entry point is through the website, using frequent status updates to pinpoint exactly the tasks currently needed.

The two cases approach collaboration and progress in different ways. TSLS takes a very transparent approach, posting raw data, containing the discussion to publicly-available websites and placing results in the public domain. CSIR OSDD takes a more cautious approach with a significant amount of work being performed through face-to-face or Skype meetings, greater use of private e-mail exchanges and a license that emphasizes mostly trust in the project's mission rather than legally-binding clauses stipulating open access to the data.

Funding was an absolute necessity for both projects. Without it, they would not have been able to access the laboratories and physical supplies needed for drug innovation, hire the minimum number of employees needed to give the projects their initial momentum, or perform routine administrative functions (such as website hosting). How much savings each project has achieved through the use of volunteers is uncertain. The Global Alliance for TB Drug Development calculated in 2001 that the estimated costs of discovering and developing a new anti-tuberculosis drug (including the costs of failure) where between US$115 million and US$240 million [58]. CSIR OSDD has about US$35 million at its disposal but they are still in early days, having yet to embark upon the most expensive part of the process, clinical trials. Maurer [59] in 2005 estimated that lead compound optimization costs between millions to tens of millions of US dollars. The chemists from TSLS achieved their result with about US$330,000. However, they were working with a known, effective lead compound with a specific problem.

The results of this case study cannot be generalized to all open source drug discovery projects since we only examined two, separate efforts. However, we believe that our findings are relevant to other projects interested in the open source model. Firstly, the cases give an indication of the number of participants necessary to achieve different drug discovery tasks. TSLS managed to complete its task with a relatively modest 37 individuals, with only a few of these dedicating large amounts of time to the project. On the other hand CSIR OSDD will require hundreds of contributors to discover and develop a new tuberculosis medicine.

The market realities of the potential drugs may also play a role in a project's adherence to the strict definition of open source. CSIR OSDD has reasonable grounds for protecting their data through a gated community and a protective license, namely that new tuberculosis products offer private companies with a profit potential in both developed and developing countries, especially lucrative if they have not had to invest in R&D. The public domain is not actually an intellectual property right but the *absence* of one. If a patent were to be granted to others on the knowledge developed by CSIR OSDD, the only way to defend against that claim would be a costly court trial. One can therefore argue that CSIR OSDD has utilized a protective license as a negative measure to safeguard others trying to patent the knowledge. In contrast the risk that TSLS' version of praziquantel will be expropriated and patented is next to null since schistosomiasis is only endemic to developing countries and the generic form of praziquantel is already available cheaply.

Unfortunately our case study is weakened by a rather low interview response rate from the CSIR OSDD project. We surmise that our timing was unlucky as an article critical of the project appeared just before we started recruitment [60]. This paper criticized the project for not publishing its first results in a peer-reviewed journal. A few potential interviewees expressed skepticism that we did not harbor an ulterior, negative motive. We debated the benefits of offering a cash prize to gather additional respondents but decided that this may only fuel the skepticism surrounding our study. We attempted to compensate by closely examining the content on the websites including interactions and self-reported data. We also submitted our results to the project manager of each of the two cases and incorporated their feedback into the final paper.

Are CSIR OSDD and TSLS model cases for open source drug discovery? It is too early to tell. Since there are so few instances of open source drug discovery, the model is still being developed, most recently with an interesting new joint project between TSL and CSIR OSDD with a focus on malaria initiated in 2011 [61]. More modeling is still needed, especially in evaluating the potential of hybrid models that combine open source with standard intellectual property mechanisms like data exclusivity and secrecy. Interesting examples (like the public-private partnership, the Archipelago to Proof of Clinical Mechanism [62]) are combining these approaches in the areas of neurology and oncology.

The recently released report of WHO's Consultative Expert Working Group on R&D Financing and Coordination [5] has called for greater use of “open knowledge innovation”. This concept is more general than open source and groups open source drug discovery with equitable licensing, patent pools and prizes (in other words, creating a grouping of the drug discovery and access business models with a primary focus of sharing of open knowledge, particularly to meet the needs of low-income countries). As organizations consider acting on the expert group's recommendations, and possibly funding organizations begin requiring a certain level of adherence to the open source model, the model will become more mainstream, giving a new level of transparency and access to the data needed to more efficiently finding cures for neglected diseases.

## Supporting Information

Annex S1 Interview template.
(DOC)

Annex S2 A survey of potential members of the CSIR OSDD project.
(DOC)
