# Supplementary material for: Open Source Drug Discovery in Practice: A Case Study
Source: PLoS Negl Trop Dis. 2012 Sep 20;6(9):e1827. doi: 10.1371/journal.pntd.0001827 (PMC3447952; doi:10.1371/journal.pntd.0001827)
Supplement: Annex S1 — Interview template. (DOC) [file pntd.0001827.s001.doc]

**Annex 1 – Interview template**

Background information

1. What academic degrees do you hold and in what subjects?
2. What is your main profession (or if you are a student, what profession do you aspire to be)?
3. What is your current professional status (for example, student, employed, self-employed, unemployed or retiree)?
4. How did you find out about Project X?
5. Why do you participate in Project X?
6. How long have you participated in Project X?
7. How much time in an average week do you spend reading or working on tasks for Project X?
8. Are you paid to spend time reading or working on tasks for Project X? Does your employer have policies that permit or encourage open source involvement? What benefits, if any, does your employer hope to gain from your involvement in this project?
9. What tools and/or resources must an individual have access to in order to contribute to Project X?
10. When a task requires laboratory work, how do you manage to gain access to the laboratory? Is payment required and if so, who pays?
11. When a task requires the use of physical resources (e.g. chemicals, etc.), how do you manage to secure the resources and who pays for them?

Collaboration

1. Do you perform tasks for Project X individually or as a part of a team? If as part of a team, please describe how you have joined the team and how you share responsibilities. How does the team communicate with the Community, for example do you have a single leader who speaks for the group or do all of you contribute directly?
2. When you are working on a task for Project X, are you able to receive feedback on your work from other members of the community? Please describe.
3. When a contribution is made, how is it received by the community? What type of review is performed?
4. How are group communications documented? Can they be accessed from archives?
5. How and why did you choose the tasks that you have performed for the community?
6. Are your tasks independent from other members’ tasks? Or are you dependent upon the results of others in order to complete your tasks? Have you built on other members’ work? Please describe.
7. Do you feel time pressure to complete your tasks? For example, are there deadlines for your tasks? Who sets them? How realistic are they? What happens if you miss a deadline?

Intellectual Property Management

1. Content posted to Project X is subject to License X. Have you read the license? Do you feel that it is important to be familiar with this license? Did you consult your employer before accepting this license? Has your employer limited the work you can do because of this license?
2. What do you think are the benefits and disadvantages of this license?
3. What is your understanding of the public domain? Do you believe that public domain status will make people more or less willing to join the community? Why or why not?
4. Have you published or considered publishing findings obtained in the course of your work for the community? If so, has publication affected the timing/content of your postings in any way? Please describe.

Progress and Commercialization

1. Has the community made progress in developing X? How has the open source model facilitated or hindered progress to date? Please describe and provide examples.
2. How is performing research and development through Project X different from other research environments (academic, student, commercial) that you may have participated in the past? Which research model(s) do you prefer from a personal standpoint? Which research model(s) are likely to be the most efficient? Please describe.
3. Please describe the remaining milestones necessary to bring product X to market? Who will perform this work? Who will provide the resources?
4. What lessons have you learned from this project? What would you do the same or differently if you were asked to organize a new open source drug discovery project?
