# Supplementary material for: Open Source Drug Discovery in Practice: A Case Study
Source: PLoS Negl Trop Dis. 2012 Sep 20;6(9):e1827. doi: 10.1371/journal.pntd.0001827 (PMC3447952; doi:10.1371/journal.pntd.0001827)
Supplement: Annex S2 — A survey of potential members of the CSIR OSDD project. (DOC) [file pntd.0001827.s002.doc]

**Annex 2 - A survey of potential members of the CSIR OSDD project**

1. Prior to today, were you aware of the online genomics map of Mycobacterium tuberculosis called TBrowse? (Yes, No, I don’t know, Other)
2. Have you ever viewed the TBrowse genome browser? (Yes, No, I don’t know, Other)
3. How useful did you find TBrowse for your research? (Not useful, Slightly useful, Useful, Moderately useful, Extremely useful, Not applicable)
4. Do you plan to use TBrowse again? (Yes, No, I don’t know, Other)
5. Why have you not used the TBrowse genome browser? (Free text)
6. Would you consider uploading your own data to the TBrowse genome browser? (Yes, No, I don’t know, Other)
7. Why would you consider uploading your own data to TBrowse? (Free text)
8. Why would you not consider uploading your own data to TBrowse? (Free text)
9. Do you have any comments regarding the TBrowse genome browser? (Free text)
